# The Importance of Cognitive Phenotypes in Experimental Modeling of Animal Anxiety and Depression

**DOI:** 10.1155/2007/52087

**Published:** 2007-07-30

**Authors:** Allan V. Kalueff, Dennis L. Murphy

**Affiliations:** Laboratory of Clinical Science, National Institute of Mental Health, Bethesda, MD 20892-1264, USA

## Abstract

Cognitive dysfunctions are commonly seen in many stress-related disorders, including anxiety and depression—the world's most common neuropsychiatric illnesses. Various genetic, pharmacological, and behavioral animal models have long been used to establish animal anxiety-like and depression-like phenotypes, as well as to assess their memory, learning, and other cognitive functions. Mounting clinical and animal evidences strongly supports the notion that disturbed cognitions represent an important pathogenetic factor in anxiety and depression, and may also play a role in *integrating* the two disorders within a common stress-precipitated developmental pathway. This paper evaluates why and how the assessment of cognitive and emotional domains may improve our understanding of animal behaviors via different high-throughput tests and enable a better translation of animal phenotypes into human brain disorders.

## 1. INTRODUCTION

**Figure 1:**
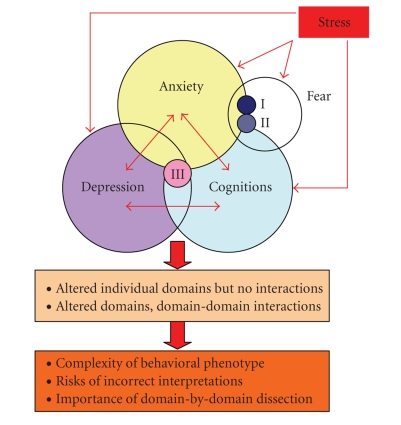
Interplay between fear, anxiety (including posttraumatic stress (I), and phobic disorders (II)), depression (including recurrent depression associated with negative memories (III) and cognitive domains in experimental models of neuropsychiatric disorders.

Cognitive processes play a key role in stress-related neuropsychiatric disorders, including emotional disorders such as anxiety and depression [1–5] (Figure 1). Abundant clinical and animal evidences strongly support this notion, suggesting that disturbed cognitions per se are an important part of affective illnesses, helping integrate the two disorders within a common stress-precipitated pathogenesis [6–10]. Indeed, strong negative memories play a key role not only in different subtypes of anxiety (especially in post-traumatic stress disorder or specific phobias) [6, 11–14], but also in depression and suicidality [15–20]. These findings are further supported by recent data from psychiatric genetics [2, 21–25] and brain imaging [26–29], showing how altered cognitions, associated with genetic contributions and inherited brain anatomy and physiology traits, modify emotional regulation of stress, anxiety, and depression.

Animal experimental models of brain disorders are an indispensable tool in today's biomedical research [5, 30–32]. Animal memory-anxiety and memory-depression interplays, as well as the genetics, pharmacology, and neurophysiology of this interplay, have been comprehensively evaluated in several reviews [33–36], further strengthening the importance of memory assessment in behavioral phenotyping [37–41].

Do we routinely do this? Clearly not, as there exist several objective and subjective reasons. First, there is a traditional dichotomy between “emotional” domains (such as anxiety and depression) and “cognitive” domains (such as memory and learning) in behavioral neuroscience. Albeit relatively artificial, these boundaries somehow seem to preprogram researchers, who often enter (and remain loyal until the retirement party) the field as either “stress scientists” or “memory researchers.” While some inquisitive scholars may subsequently move from one “cast” to another during their careers, in many cases it is the initial professional choice, triggered by personal preferences and reinforced by age-dependent conservatism, that dictates the whole line of subsequent behavioral research of a scientist. Sadly, such heterogeneity often further divides behavioral neuroscientists, who sometimes tend to attend only specialized meetings within their “own” domains, concepts and paradigms.

Another reality is that “anxiety” or “depression” laboratories rather rarely study memory and learning phenotypes in depth (and vice versa), and do so mostly when a gross cognitive deficit is apparent and seems to influence all outgoing animal behaviors. In many such cases, memory testing becomes rather formal, is limited to selected “reference” memory tests, and does not focus on complex *interactions* between memory, anxiety, and depression domains (see, however, several encouraging exceptions discussed further).

Likewise, despite a growing recognition of the deleterious consequences of restricted behavioral battery usage [42, 43], current routine problems of an average behavioral laboratory include limits in testing and animal holding space, the lack of proper behavioral training, personnel, limited research budgets, or all of them together. Collectively, this leads to an extensive use and reuse of animals in high-throughput batteries [44–46]. In reality, this means that emotionality (e.g., anxiety and depression) tests are routinely run in the same cohorts of animals with relatively little attention to possible cognitive mechanisms or alterations that are triggered by such batteries, and that may, in fact, influence dramatically the subsequent behavioral scores of “anxiety” and “depression” [44]. Furthermore, learning and memory per se may also be affected by such batteries [44], further complicating behavioural phenotyping, and most likely exerting secondary effects on anxiety and depression.

Is this of concern? Can our routine laboratory practice lead to confounded findings and, even worse, potential misinterpretations of data? The aim of this paper is to analyze why and how an in-depth assessment of cognitive and emotional domains may improve our understanding of animal behaviors in different high-throughput tests, and their translation into human behavioral disorders.

## 2. TARGETING MEMORY-ANXIETY INTERPLAY IN ANIMAL BEHAVIORAL MODELS

Learning, memory, and anxiety have long been known as interactive dimensions in both animal and clinical studies [47, 48]. The importance of in-depth assessment of memory and anxiety together is further illustrated in Table 1. The interplay of these two domains in this table may hypothetically lead to multiple alternative states, whose misinterpretations in different behavioral tests (as well as psychopharmacological data obtained in such models) would generally be unavoidable if only single domains were assessed (also see: [31, 32] for discussion). In a similar vein, a recent review [41] has evaluated anxiety and memory/learning phenotypes in various genetically modified mouse models, including mutant mice lacking various receptors or other brain proteins. A common (but not mandatory) situation noted in this study, when the same mutation leads to simultaneously altered anxiety and memory phenotypes, illustrates the overlap between these two key domains, and demonstrates the extent to which their interplay may affect other animal outgoing behaviors.

In fact, some of phenotypes that we do observe in different models strikingly parallel hypothetical situations modeled in Table 1 (see, for example, altered anxiety and cognitions in 5-HT1a and 5-HT1b receptor knockout mice, and the ways to dissect their possible interplay, in [30–32]). Adding further complexity to the problem, it is always important to consider potential heterogeneity of memory subtypes, as the same mutation (such as 5-HT1b receptor knockout) may impair one type of memory (e.g., habituation) while improving another (e.g., spatial memory) [30].

Several other interesting directions of research may be considered further, based on specific targeting of memory-anxiety interplay. For example, as some subtypes of anxiety problems, such as post-traumatic stress disorder (PTSD), are based on strong aversive memories, genetic and behavioral models with both high anxiety and memory components [41, 49, 50] may lead to more valid experimental models of PTSD. However, some difficulties may also be likely with such models, as PTSD-like hyperarousal, commonly observed both clinically and in animals [49], may possibly be misinterpreted as increased locomotion (suggestive of anxiolytic-like phenotype). In any case, researchers should be aware of such interpretational difficulties, and make their conclusions with necessary caution and after testing several alternative hypotheses (see Table 1 for examples).

Finally, genetic models may target reciprocal interplay between these domains that are potentially relevant to mechanisms of stress resistance. Likewise, mice with both reduced anxiety and memory (see [41] for review) may lead to genetic models focused on mechanisms of resistance to PTSD and other types of anxiety associated with recurrent negative cognitions (see [6, 47]).

## 3. MODELING MEMORY-DEPRESSION INTERPLAY

The importance of cognitive mechanisms in clinical depression has long been known in the literature [51]. Indeed, we need to remember our past traumas and frustrations in order to become properly depressed. Memory and learning have also been considered in animal models of depression (e.g., see[52]). How can we apply this understanding to our experimental models and do it correctly? Table 2 summarizes a hypothetical situation where two interplaying domains (depression and memory) may lead to multiple alternative states, whose misinterpretations in different behavioral tests seem to be highly likely.

Some interesting experimental models of neuropsychiatric disorders may arise from specific targeting of memory-depression interplay. For example, since recurrent intrusive negative memories frequently accompany clinical depression [53–56], animal models based on simultaneously increased memories and depression-like phenotypes [52, 57–59] may be clinically relevant to modeling affective disorders associated with negative cognitions. In contrast, mouse models with cooccurring memory deficits and reduced depression-related behaviors (such as 5-HT1a knockout mice, see [60]) may be potentially useful to understand mechanisms of resistance to depression associated with chronic negative memories [61].

## 4. MODELING WITHIN AND BEYOND

With recent strategies of behavioral modeling of anxiety and depression (see [62]) supporting expansion beyond “pure” anxiety and depression domains, experimental models based on targeting these plus cognitive domains represent further important directions of research. One strategy may be to apply more extensively the models and tests that simultaneously profile anxiety (or depression) and memory functions. Conceptualized as behavioral “models-hybrids” [62, 63], this approach allows minimization of the unwanted behavioral consequences of test batteries, and provides an extensive high-throughput phenotyping of animals with a fewer number of procedures. For example, increased anxiety in the elevated plus maze and the loss of benzodiazepine anxiolytic efficacy upon repeated testing [48] may be used to indirectly assess memory functions in different mutant or drug-treated animals, as evaluated by the presence or absence of the above-mentioned “one trial tolerance” phenomenon. Likewise, the forced swim test (measuring “despair” depression domain) may be used to assess within- and between-trial habituation (spatial working and long-term memory) and *learned* helplessness. Fear conditioning, including active avoidance tests [64, 65]) are highly relevant to both fear (anxiety-related) and cognitive (learning) domains. Y- and T-mazes allow parallel assessment of spatial memory, exploration (anxiety), and spontaneous alternation. Morris water maze, a traditional hippocampal memory test, can also be used to study depression-like traits (e.g., immobility in [66, 67]). Finally, various elevated mazes can be used to profile cognitive domains (memory, learning) as well as animal anxiety [68, 69].

In general, there may be other combinations of anxiety, depression and memory tests, or even more sophisticated hybrid models, that could be used more extensively for high-throughput behavioral phenotyping. However, another reason to use these models more widely in behavioral research is the possibility of performing an *integrative* (versus more traditional, domain-oriented) experimental modeling of brain disorders. This approach, based on targeting commonalities (rather than differences) of disorders, will allow researchers to parallel their animal models with recent trends in clinical psychiatry, where “continuum” or “spectrum” theories are beginning to challenge the existing “heterogeneous” Kraepelinian paradigms [70–72].

An important step in this direction may be the use of rodent models that simultaneously evaluate “comorbid” anxiety and depression and also focus on cognitive (dys)functions in these models. For example, selectively bred HAB mice [52] and thyroid hormone receptor knockout mice [9] display inherited anxiety- and depression-like phenotypes, and their cognitive functions merit further studies (see, e.g., aberrant memory in the latter model). Similarly, olfactory bulbectomy, traditionally known to produce depression in rodents, has been recently reported to be relevant to comorbidity of anxiety and depression, and is accompanied by specific memory deficits in animals that resemble cognitive dysfunctions in humans with comorbid anxiety and depression [5].

Further important information can also be obtained through in-depth ethological analyses of behavioral strategies, including cross-species and cross-strain comparisons [73, 74] of animal behaviors in different tests—an approach consistent with recent endophenotyping and cross-species trait genetics concepts in animal behavioral modeling [75, 76]. Finally, expanding far beyond anxiety and depression domains may also be a rational strategy of research, as it allows modeling of complex schizo-affective and neurodevelopmental disorders based on increased anxiety, depression and altered memory, and other cognitions [77–80].

## 5. CONCLUDING REMARKS

To optimize behavioral phenotyping research, the neuroscientific community may need to encourage behavioral neuroscientists to produce data on memory and learning phenotypes in their papers that report anxiety- and depression-related behaviors (e.g., [30, 31, 60]). As a practical solution, “can my findings be a result of merely altered memory or learning?” should be one of the first questions asked in studies on animal emotionality and affective behaviors. In cases when both cognitive and emotionality domains seem to be affected (e.g., [81, 82]), we next need to establish the nature of their interactions, and how they might codetermine the behavioral phenotype observed. Finally, in addition to studying behavior x gene x environment interactions, we may benefit from focusing on behavior x cognitions x gene x environment interactions. “Work hard and marry a talent”—advised R. Blanchard in one of his interviews, sharing with fellow colleagues the recipe for a successful career in science. Following such wise advice, diligent behavioral neuroscientists working with anxiety and depression may benefit from joining forces with (and even perhaps marrying) their talented colleagues studying memory and learning.

## Figures and Tables

**Table 1:** Examples of possible interplay between memory and anxiety domains, and how this may lead to misinterpreted animal behavioral and drug-induced phenotypes (effects: ↑ increased, ↓ reduced behavior). Note that real animal models have multiple other factors and domains, and the complexity (and risks of incorrect interpretation) of their phenotypes is much higher.

Domains	Anxiety		
Memory, learning	Elevated	Unaltered	Reduced
Elevated	Likely phenotype: ↑ initial anxiety (↓ activity) with ↑ habituation (anxiolytics would ↓ hypoactivity and habituation). Possible misinterpretation of baseline phenotype: hyperanxiety; ↓ sensitivity to repeated stressors (while, in fact, having ↑ vulnerability to chronic stress)	Likely phenotype: ↑ habituation [anxiolytics would ↑ activity and ↓ habituation]. Possible misinterpretation: ↓ exploration (↑ anxiety). Anxiolytics would ↓ habituation (however, this may be mistaken for ↓ anxiety)	Likely phenotype: ↓ initial anxiety with ↑ habituation (anxiolytics would ↓ habituation) Possible misinterpretation: initial hyperactivity followed by ↑ freezing (“↑ anxiety”). Anxiolytics will ↓ habituation (however, this may be mistaken for mild psychostimulant action)

Unaltered	Likely phenotype: ↑ anxiety (↓ exploration), normal memory. Anxiolytics may ↓ anxiety and memory. In some tests phenotype may be misinterpreted as baseline hypolocomotion		Likely phenotype: reduced anxiety (↑ exploration), normal memory. Anxiolytics may impair memory without affecting (already low) anxiety In some tests baseline phenotype may be misinterpreted as hyperactivity

Reduced	Likely phenotype: ↑ initial anxiety with ↓ habituation. Anxiolytics may ↓ anxiety and further impair memory. Possible misinterpretation of baseline phenotype: hypersensitivity to repeated stressors (while, in fact, having ↓ vulnerability to chronic stress). Effects of anxiolytics may be mistaken for psychostimulant action	Likely phenotype: ↓ habituation. Anxiolytics may further impair memory. Possible misinterpretation of baseline phenotype: ↑ exploration (↓ anxiety). Effects of anxiolytics may be mistaken for psychostimulant action	Likely phenotype: ↓ initial anxiety with ↓ habituation (anxiolytics may ↓ memory). In some tests may be misinterpreted as persistent hyperlocomotion. Effects of anxiolytics may be mistaken for psychostimulant action

**Table 2:** Examples of possible interplay between memory and depression domains, that may lead to misinterpreted animal behavioral phenotypes (effects: ↑ increased, ↓ reduced behavior; OCD-obsessive-compulsive disorder). Given high research pressure on behavioral labs, consider the likelyhood of incorrect interpretation of behavioral data.

Domains	Depression		
Memory, learning	Elevated	Unaltered	Reduced
Elevated	Likely phenotype: hypoactivity (or stereotypic hyperactivity in some tests) but ↑ sensitivity to repeated stressors. Possible misinterpretation of baseline phenotype: ↑ anxiety/freezing (or ↓ habituation, spatial memory in acute stress models)	Likely phenotype: ↑ habituation and ↑ sensitivity to repeated stressors. Possible misinterpretations: ↓ exploration (↑ anxiety) and ↑ despair depression	Likely phenotype: active locomotion with ↑ habituation and sensitivity to repeated stressors. Possible misinterpretations: initial hyperactivity followed by gradually ↑ anxiety, or ↑ “despair” depression (which, in fact, reflects ↑ learning)

Unaltered	Likely phenotype: ↓ hypoactivity (or stereotypic hyperactivity in some tests). Possible misinterpretation: ↑ anxiety/freezing (or ↓ habituation, spatial memory)		Likely phenotype: active locomotion. Possible misinterpretation of this phenotype: no or ↓ anxiety

Reduced	Likely phenotype: marked sustained hypoactivity (or stereotypic hyperactivity) with ↓ habituation and sensitivity to repeated stressors. Possible misinterpretations: ↑ anxiety (and/or OCD-like behavior) or ↓ despair depression	Likely phenotype: ↓ habituation. Possible misinterpretation: ↑ exploration (↓ anxiety)	Likely phenotype: active locomotion with ↓ habituation and sensitivity to repeated stressors. In some tests this may be misinterpreted as persistent hyperlocomotion
